# Design and Fabrication of Micro/Nano Sensors and Actuators

**DOI:** 10.3390/mi15060674

**Published:** 2024-05-22

**Authors:** Weidong Wang, Ruiguo Yang, Min Liu

**Affiliations:** 1 School of Mechano-Electronic Engineering, Xidian University, Xi’an 710071, China; mliu_12@stu.xidian.edu.cn; 2 Department of Mechanical and Materials Engineering, University of Nebraska-Lincoln, Lincoln, NE 68588, USA; ryang6@unl.edu

A micro-electromechanical system (MEMS) is a micro device or system that utilizes large-scale integrated circuit manufacturing technology and microfabrication technology to integrate microsensors, micro-actuators, microstructures, signal processing and control circuits, power supplies, and communication interfaces into one or more chips [1]. There are many types of MEMS devices, mainly including MEMS sensors [2], MEMS converters [3], and MEMS actuators [4]. MEMS sensor components are used to detect various physical properties, such as pressure [5], temperature [6], acceleration [7], and angular velocity [8]. MEMS converters convert electrical signals into mechanical motion or vice versa, including the conversion of sound to electrical signals, and pressure to electrical signals [9]. MEMS actuators, integral to the functionality of MEMS devices, are engineered to facilitate precise mechanical movements at a microscale. They find application in a variety of domains, such as micromotors [10] and micro-valves [11].

MEMS devices are crafted from a variety of materials such as silicon, metal, ceramics, and glass, with the mechanical, electrical, and magnetic properties of these materials significantly influencing the operational performance of MEMS devices [12,13,14,15]. The mechanical strength and stiffness of materials determine the reliability and stability of devices [16]. Silicon materials have excellent mechanical and processing properties and are commonly used in the manufacturing of sensors and actuators [17]. Polymer materials or metal films are commonly used for making flexible MEMS devices [18]. The electrical properties of materials determine device attributes such as resistance, capacitance, and inductance, which directly affect its application in circuits [19]. Furthermore, the magnetic properties of materials are essential for specialized MEMS devices such as magnetic sensors and actuators. Tailoring these magnetic properties can significantly boost the sensitivity and stability of sensors, and enhance the precision and response time of actuators [20]. In summary, optimizing the properties of these materials can improve the accuracy, sensitivity, and reliability of MEMS devices, thereby expanding their application scope in various fields.

In addition, process manufacturing is critical to determine the performance and reliability of MEMS devices [21]. The accuracy and stability of process manufacturing directly affect the performance of devices [22]. The accuracy of micro/nano-processing technology determines the exact dimensions and configuration of the device structures, while the process stability of the process ensures the consistency and repeatability of the device. For example, the precise control of process steps such as photolithography, thin film deposition, and ion etching can ensure the accuracy and stability of the device structure, thereby improving the performance and reliability of the device [23,24].

This Special Issue encompasses 11 papers that explore various facets of MEMS/NEMS, including the design and optimization of MEMS devices (Contributions 1–7), micro/nano-materials of MEMS devices (Contributions 8), and micro-manufacturing processes of devices (Contributions 9–11).

In particular, Wei et al. (Contribution 1) designed a high-performance piezoelectric-type MEMS vibration sensor based on LiNbO_3_ single-crystal cantilever beams. The proposed MEMS vibration sensor has a high output performance, linear dependence, and stable sensitivity, and is suitable for broadband high-frequency vibration detection. Zhao et al. (Contribution 2) investigated the effect of the micro-morphology of resistive strain gauges on the gauge factor. The study showed that periodic indentations on the sidewalls of the sensitive grid enhance local strain concentration and weaken strain distribution on the grid body, indicating that a rough microstructure can lead to a decreased strain coefficient, thereby reducing the accuracy and sensitivity of resistive strain gauges. Chen et al. (Contribution 3) proposed a micromechanical transmitter with only one bulk acoustic wave (BAW) magneto-electric (ME) antenna. A single-BAW ME antenna can replace traditional transmitter components and adjust the radiation power of the BAW ME antenna by increasing the input voltage in higher-order resonance modes. Guo et al. (Contribution 4) designed and optimized a MEMS skin friction sensor with a high response frequency and large measurement range. The sensor was statically calibrated using the centrifugal force equivalent method and a single-axis-rotating loading platform. The sensor had good linearity and stability, and high assembly accuracy, which meets the testing requirements of hypersonic wind tunnel experiments. Ren et al. (Contribution 5) proposed a design which improved a bulk acoustic wave magnetic sensor based on magnetoelectric coupling. The material design of inserting an Al_2_O_3_ thin film layer into an FeGaB and a two-layer piezoelectric magnetic/piezoelectric heterostructure reduced the eddy current loss of the magnetic composite material and elevated the energy conversion efficiency of the sensor. Cai et al. (Contribution 6) presented an improved temperature compensation approach called proportional difference for accelerometers based on differential frequency modulation to cancel out the frequency drift caused by temperature change. A parameter named temperature difference ratio was used to cancel the drift in the frequency of the differential resonators caused by temperature. Liu et al. (Contribution 7) reviewed the research progress of inertial switches. They introduced the design concept of MEMS inertial switches, providing an overview of their performance, including sensitive direction, acceleration threshold, and contact enhancement.

To study MEMS device materials, Tian et al. (Contribution 8) investigated the hydrogen storage performance of γ-graphdiyne-doped Li based on first principles. The results indicated that doping Li atoms could enhance the hydrogen storage property of intrinsic γ-GDY when in large-capacity hydrogen storage. Additionally, vacancy defects can improve hydrogen storage performance, and Li-VGDY possesses better hydrogen storage performance than Li-GDY.

Regarding the manufacturing of MEMS devices, Lou et al. (Contribution 9) reviewed the latest advances in the preparation technologies for micro-metal coils. They discussed the typical structural types of micro-metal coils and applications, summarized the preparation materials and main preparation methods of micro-metal coils, including macroscopic preparation processes (printed circuit board (PCB) process, hand winding method, and wire welding technology), MEMS processing technology, and other manufacturing technologies. Baek et al. (Contribution 10) proposed a manufacturing process of polymeric microneedle sensors for mass production, which can be applied in the electrochemical detection of various biomarkers in interstitial fluid. The proposed manufacturing process effectively produces microneedles with high aspect ratios and different lengths, and can be replicated. Zhong et al. (Contribution 11) proposed a novel method which transfers tactile sensors by using stiction effect temporary handling (SETH). This method simplifies the microelectromechanical system (MEMS)/CMOS integration process, improves the process reliability and electrical performance, and reduces material constriction. Moreover, they introduced the principle of using SETH for CMOS compatible batch transfer tactile sensors and provided the design of temporary adhesive structures to reduce adhesion forces caused by adhesion effects. In addition, Liu et al. (Contribution 7) introduced the manufacturing methods for non-silicon surface microfabrication technology, standard silicon microfabrication technology, and liquid inertial switches.

I would like to take this opportunity to thank all the authors who have submitted their papers to this Special Issue, and all the reviewers for dedicating their time and helping to improve the quality of the submitted papers.
